# The cortisol response to exercise in young adults

**DOI:** 10.3389/fnbeh.2015.00013

**Published:** 2015-02-03

**Authors:** Henning Budde, Sergio Machado, Pedro Ribeiro, Mirko Wegner

**Affiliations:** ^1^Faculty of Human Sciences, Medical School HamburgHamburg, Germany; ^2^Sport Science, Reykjavik UniversityReykjavik, Iceland; ^3^Laboratory of Pânico and Respiration, Institute de Psychiatry, Federal University of Rio de JaneiroRio de Janeiro, Brazil; ^4^Physical Activity Neuroscience, Physical Activity Sciences Post-Graduate Program, Salgado Oliveira UniversityNiterói, Brazil; ^5^Institute de Psychiatry, Federal University of Rio de JaneiroRio de Janeiro, Brazil; ^6^Deptarment of Sport Psychology, Sport Pedagogy, and Research Methods, Institute of Sport Science, University of BernBern, Switzerland

**Keywords:** resistance exercise, cognition, cortisol, hypothalamo-hypophyseal system, young adults

Tsai et al. (2014) conducted a study related to Budde et al. (2010b) who examined the change in steroid hormones as potential biochemical mechanisms underlying the beneficial effects of acute exercise on cognitive performance. Tsai et al. (2014) investigated how young male adults between 20 and 29 years of age respond to two types of resistance exercise with a high intensity group (HI), a medium intensity group (MI), and a control group. The outcome measures were cortisol, insulin-like growth factor 1 (igf-1), and growth hormones as well as electroencephalography (EEG). One of the authors question was whether these parameters could be made responsible for a change in cognitive functioning (measured with a Go/No-Go task combined with the Eriksen Flanker paradigm). The resistance exercise consisted of approximately 40 min of high-intensity (80% one-repetition maximum, 1RM) and moderate-intensity (50%, 1RM) acute resistance exercise on the exercise machines, respectively (Tsai et al., 2014). In their paper, the authors did not mention important research findings in regard to the effect of different intensity and duration levels of an acute bout of exercise on the cortisol concentration. So, in response to exercise, the hypothalamus secretes corticotropin-releasing hormone, which activates the anterior pituitary, stimulating the release of adrenocorticotropic hormone, which in turn stimulates the adrenal cortex to release cortisol. This has been shown in numerous reports. Research in adults revealed that if a certain physical effort is exceeded the cortisol level rises as a function of both, intensity and duration (Kirschbaum and Hellhammer, 1994; Brownlee et al., 2005; Hill et al., 2008; Gatti and De Palo, 2011). Consistently, it has been shown that an exercise intensity exceeding 60% of the individual's maximal oxygen uptake (VO_2max_) induces cortisol release above resting levels in adults. A minimum duration of 10–15 min may result in increased cortisol levels with peak concentrations 20–30 min after the cessation of the exercise bout (Kirschbaum and Hellhammer, 1994). In agreement with findings in studies examining adults, adolescents show a similar reactivity of the hypothalamic–pituitary–adrenal axis resulting in cortisol increases in response to acute bouts of exercise. For 15- to 16-year-old adolescents, for example, a 12-min bout with an intensity of 70–85% of the maximum heart rate (HR_max_) led to an increase in cortisol levels contrasted to a group exercising with moderate intensity (50–65% HR_max_) (Budde et al., 2010b) or compared to a cognitive stressor, respectively (Budde et al., 2010a). A recent study investigated how cortisol levels of adolescents at the age of 14 react to different stressors (Wegner et al., 2014). In this study, exercise was induced by running 15 min at a medium intensity level of 65–75% HR_max._. Other than the psychosocial stressor, the acute bout of physical exercise was not able to significantly increase cortisol levels.

These results confirm the threshold phenomenon for adults also in adolescents of late puberty stages and indicate that the concentration of cortisol after acute bouts of exercise is intensity dependent. In contrast to these results Tsai et al. (2014) found that post-exercise concentrations of cortisol were significantly lower than the pre-exercise ones in both groups. Tsai et al. (2014) argued their findings would be in line with the results showing cortisol concentrations to be decreased immediately post-exercise and for up to 1–2 h post-exercise compared to pre-exercise (Kemmler et al., 2003; Heaney et al., 2013). However, both studies investigated completely different age groups. Kemmler et al. (2003), for example, dealt with early postmenopausal women (mean age 56.4) while Heaney et al. (2013) conducted their research with elderly males and females between 60 and 77 years of age. Heaney et al. (2013) achieved their findings using incremental submaximal treadmill exercise lasting on average 23 min with participants reaching 76.5% of their predicted HR_max_. They interpreted their findings carefully in the light of the majority of studies showing a rise in cortisol, even in this age group. Therefore, Heaney et al. (2013) formulated a restriction: Because the exercise was graded, it is possible that participants did not exercise for a sufficient period at 60% VO_2max_ or above to elicit an increase in cortisol. Kemmler et al. (2003) used acute endurance exercise at 75–85% HR_max_, 5 min of jumping exercises (65–85% HR_max_), and 45 min of intense strengthening (25 sets, ten repetitions at 75% 1RM, 1 min of rest). They limited their findings by suggesting that the diurnal timing of the exercise session may be partially responsible for the observed decrease in cortisol levels. The 36% decrease during the first sampling interval (7:45–9:05 a.m.) was significantly higher than the 14% decrease during the longer second interval (9:05–11:00 a.m.). Thus, the reported cortisol data in the paper by Tsai et al. (2014) lack support from the literature and are unusual and far lower than one would expect. Different methodological shortcomings may have led to these contrasting results. One possible factor, which is discussed by Tsai et al. (2014) is the daytime of testing. Another potential influencing factor is the timing of measurements. Specific to cortisol, Daly et al. (2004) reported that the time-point at which blood samples are collected with respect to exercise can greatly affect the interpretation of data outcomes. A topic that is especially interesting because in their design Tsai et al. (2014) waited for participants' body temperature and HR to return within 10% of pre-exercise levels, prior to taking a blood sample (it is also stated that additional blood samples were taken after the subjects had completed the cognitive task but the exact time frame remains unclear). Another problem is the different measurement techniques in the referred studies (saliva vs. blood; see Gatti and De Palo, 2011). Most of these possible explanations for the contradicting results are lacking and the data should thus be interpreted with caution. In the future it is important to state more clearly what the specific time of day was, when procedures were carried out, considering that testing time may alter the interpretation of the results, especially for the impact on cognition.

## Conflict of interest statement

The authors declare that the research was conducted in the absence of any commercial or financial relationships that could be construed as a potential conflict of interest.
